# Health Tract, No. 263

**Published:** 1867-03

**Authors:** 


HEALTH TRACT, No. 263.

### EXPERIMENTING.

THE man's horse is said to have died on the very day the owner had come to the conclusion that he had succeeded in the experiment of teaching him how to live without eating.

Mr. William Buston of Wisconsin, a bachelor of forty-five, had been experimenting for some time in subjecting himself to great heat as a means of rejunevation and of bringing back that supleness of limb, that fullness of flesh, that buoyancy of spirits which he remembered to have posessed a quarter of a century earlier. One morning he was found in his room perfectly lifeless, the heat he had subjected himself to having been too intense for mere flesh and blood.

Some girls have induced fatal ailments by drinking large quantities of vinegar under the supposition that it would diminish the quantity of superfluous flesh, which seemed to be their affliction. Others have taken large quantities of tea, by chewing it.

City physicians are very frequently brought in communication with persons who, from some whim or caprice, have adopted modes of life the very reverse of what an intelligent judgement would have dictated.

Citizens in imperfect health often go to the country with extravagant anticipations of amendment—that living on pure milk and fresh eggs will accomplish wonders, not knowing that milk is the natural food of babies, kittens, and the like. Grown persons who use sweet milk largely every day, invariably become bilious or constipated, unless steady, hard labor in the open air every day, allows its use with impunity.

A lawyer — a man of family, then a canidate for legislative honors — was suffering greatly from constipated habits and had been experimenting on the theory for sometime that the best way of effecting an ejectment was to eat heartily and push it out! He was evidently sincere in his representations to us.

Most men would hesitate to attempt the repair of a watch or even an old shoe, and yet not a few are found to experiment in the repair of the most intricate yet beautiful machinery of human life without apparent fear of harm. Those who plead their own cases in court and those who attempt to tinker up their own constitutions are equally unwise.

If your pants need a patch, send for a tailor; if your body is out of order, consult a physician.

